# A predictive model for body water and fluid balance using 3D smartphone anthropometry

**DOI:** 10.3389/fphys.2025.1577049

**Published:** 2025-06-23

**Authors:** Austin J. Graybeal, Abby T. Compton, Sydney H. Swafford, Caleb F. Brandner, Molly F. Johnson, Maria G. Kaylor, Hunter Haynes, Jon Stavres

**Affiliations:** ^1^ School of Kinesiology and Nutrition, University of Southern Mississippi, Hattiesburg, MS, United States; ^2^ Department of Health and Human Physiology, University of Iowa, Iowa City, IA, United States

**Keywords:** anthropometry, body composition, 3D, body fluid, water retention, fluid balance, overhydration, edema

## Abstract

**Background:**

Body fluid volumes, including total body water (TBW), extracellular fluid (ECF), and intracellular fluid (ICF), are crucial indicators of body composition, and the distribution of these fluids is essential for assessing hydration status and fluid accumulation. Although fluid volumes are commonly measured with bioelectrical impedance devices, several challenges hinder the application of this technique. However, 3D smartphone scanning applications that automate body volumes and other anthropometric estimates may provide a viable alternative to body fluid assessments.

**Methods:**

A total of 338 participants underwent fluid volume assessments using bioelectrical impedance spectroscopy (BIS) and collected body volumes and anthropometric data using a 3D smartphone scanning application. Then, LASSO regression was used to develop new TBW and ECF prediction model in a subset of participants (n = 272), which was subsequently tested in the remaining participants (n = 66). Smartphone-derived ICF was calculated as the difference between smartphone-predicted TBW and ECF. Fluid overload and imbalance were determined using ECF/TBW and ECF/ICF, respectively, and subsequently predicted from the retained variables using receiver operating characteristic curve analyses and logistic regression.

**Results:**

Estimates from each of the newly-developed prediction models were not significantly different from the estimates produced using BIS (all p ≥ 0.70) and revealed acceptable agreement (TBW: *R* ^2^ = 0.91, RMSE = 3.24 L; ECF: *R* ^2^ = 0.94, RMSE = 1.10 L; ICF: *R* ^2^ = 0.87, RMSE = 2.29 L) when evaluated in the testing sample (n = 66), although proportional bias was observed (p < 0.001). Smartphone-predicted fluid overload (AUC: 0.81 [95%CI: 0.70, 0.92]; sensitivity + specificity: 1.53 [95%CI: 1.39, 1.67]) and imbalance (AUC: 0.76 [95%CI: 0.64, 0.88]; sensitivity + specificity: 1.40 [95%CI: 1.24, 1.56]) demonstrated acceptable diagnostic performance.

**Conclusion:**

Smartphone scanning applications can accurately assess body fluid volumes and imbalances, presenting new possibilities for health screening beyond clinical environments.

## 1 Introduction

Fluid balance assessments are a critical part of health screening and monitoring, particularly for older adults or individuals with developing cardiovascular, renal, or hepatic pathologies (Roumelioti et al., 2018). For instance, older adults are more prone to reduced kidney function and musculoskeletal atrophy, and assessing fluid balance can provide valuable insights that may help in preventing the onset of dehydration and malnutrition-related complications (Powers et al., 2009; Tanigu et al., 2017). For conditions such as heart failure and kidney and liver disease, regular monitoring of fluid volume and distribution is crucial in preventing complications like fluid overload and imbalance, which may further aggravate such conditions (Hara et al., 2009; Nongnuch et al., 2015; Hür et al., 2014; Accardi et al., 2021; Horino et al., 2023; Kim et al., 2017). While the clinical utility of these assessments is clear in specific populations, abnormal body fluid may also be indicative of worsening conditions more commonly encountered in the broader population, such as obesity and hypertension, that often precede more serious health issues (Roumelioti et al., 2018; Chang et al., 2016; Cianci et al., 2006). Regular fluid balance assessments may also provide valuable insights beyond specific pathologies, with practical applications in sports science (e.g., performance optimization, prevention of heat-related illness), general health monitoring, and thermoregulation (e.g., body temperature regulation during extreme environmental conditions).

While often used to estimate body composition parameters such as body fat percentage or fat-free mass ([FFM] standalone or within multi-compartment models), assessing fluid balance typically involves the direct or indirect volumetric measurement of total body water (TBW), which constitutes approximately 45%–60% of an individual’s body mass, and the distribution of that fluid across the extracellular (ECF) and intracellular fluid (ICF) compartments (Bhave and Neilson, 2011). Isotope dilution methods offer the most direct measurement of TBW and its distribution across cellular membranes, but the complexity of this approach limits its utility outside of research settings (Chan et al., 2008). Alternatively, non-invasive bioelectrical impedance analyses (BIA) are more commonly used, as they have been shown to provide accurate and reliable estimates that agree with dilution techniques (Moon et al., 2009; Moon et al., 2008; Cataldi et al., 2022; Tinsley et al., 2023; Martinoli et al., 2003). However, due to costs, the need for additional equipment, and the rigorous pre-assessment standardization requirements of BIA, the adoption of this technique in routine care outside of more specialized practice remains limited. Simple anthropometric measurements are often used to predict TBW, though their oversimplified nature limits their precision and their ability to distinguish between ECF and ICF levels, which are crucial for evaluating fluid overload and imbalance (Chamney et al., 2007). Therefore, there is a critical need for accessible, cost effective, and user-friendly anthropometric tools that can accurately estimate body fluid without requiring additional equipment.

Mobile digital anthropometrics, automated through three-dimensional (3D) smartphone scanning applications, may offer a potential solution. Initially used to generate 3D representations and automate hundreds of body composition and anthropometric estimates from 2D images, mobile digital anthropometrics are continuously expanding their clinical utility. Recent studies have demonstrated that the estimates produced by these smartphone scanning applications can be used in clinical settings to evaluate body image distortion and dissatisfaction (Braun-Trocchio et al., 2023; Graybeal et al., 2023a), metabolic syndrome (Medina Inojosa et al., 2024; Graybeal et al., 2024a), and, more recently, bone mineral content (BMC) (Graybeal et al., 2024b). Moreover, this technique has not only demonstrated validity against multi-compartment body composition models (Graybeal et al., 2022a; Graybeal et al., 2022b), but the body volume estimates automated by these smartphone scanning applications have also shown agreement with more invasive methods such as underwater weighing (Fedewa et al., 2021), supporting their use as a valid non-invasive proxy for body volume within multi-compartment models (Sullivan et al., 2022). Given the well-established interplay between body volume and fluid estimates (Nickerson et al., 2022), as well as the surging clinical capabilities of this technique, 3D smartphone anthropometry may be equipped to provide accurate estimates of body water and fluid balance that improve upon the limitations of existing methods. However, the ability of this technique to provide such estimates is currently unknown. Therefore, this study aimed to determine whether smartphone-derived body volume measurements could be used to predict: 1) estimates of body water and fluid distribution, and 2) fluid overload and imbalance classifications. We hypothesized that the smartphone-based body fluid prediction models would accurately estimate TBW, ECF, and ICF, and effectively distinguish fluid overload and imbalance classifications with acceptable accuracy.

## 2 Materials and methods

### 2.1 Participants

A total of 338 male and female (148 M, 190 F) participants between the ages of 18 and 60 (age: 23.8 ± 8.2 yrs; BMI: 26.0 ± 5.8 kg/m^2^) were prospectively recruited and completed this cross-sectional evaluation. Participants were excluded if they were younger than 18 or older than 60 years; were missing any limbs or part of a limb; had a pacemaker or any other electrical implant; had a substantial amount of internal metal such as a metal plate or a complete joint replacement; were diagnosed with renal, liver, or cardiovascular disease; or if they were pregnant, breastfeeding or lactating. All procedures were conducted in accordance with the Declaration of Helsinki and were approved by the University of Southern Mississippi Institutional Review Board (IRB#22-1012/23-0446). Written informed consent was obtained from all subjects prior to participation.

### 2.2 Procedures

Participants arrived at the laboratory for testing after a minimum 8-h overnight fast from food, beverage, and supplements/medications, and after abstaining from exercise for at least 24-h. Upon arrival, participants were instructed to remove any external metal or accessories (e.g., jewelry, shoes, watches, etc.) and to wear only tight, form-fitting athletic clothing (females: compression shorts/tights and a sports bra; males: compression shorts/tights only) before undergoing testing. Once all pre-assessment requirements were confirmed, participants underwent several anthropometric measurements, including height via stadiometer, weight using a calibrated scale, automated anthropometrics using a 3D body scanning smartphone application, and body fluid estimates using bioelectrical impedance spectroscopy (BIS).

### 2.3 Bioelectrical impedance spectroscopy

A comprehensive description of the BIS methods used in this study have been presented elsewhere (Graybeal et al., 2024c; Graybeal et al., 2023b; Brandner et al., 2022; Vallecillo-Bustos et al., 2024). Accordingly, the following section is presented as a summary, with specific aspects of this evaluation discussed in greater detail. A tetrapolar, hand-to-foot BIS device (SFB7; ImpediMed®, Carlsbad, CA) was used to measure body composition (body fat %, fat mass, FFM) and TBW, as well as the ICF and ECF. Importantly, body composition and fluid estimates produced by BIS have well-demonstrated agreement with criterion estimates such as dual-energy X-ray absorptiometry (DXA) and deuterium dilution, respectively (Moon et al., 2009; Moon et al., 2008; Cataldi et al., 2022; Tinsley et al., 2023; Martinoli et al., 2003; Esco et al., 2019; Earthman et al., 2007).

Prior to testing, participants were instructed to lie supine with their hands flat and to spread their hands and feet away from their body for at least 5 min to ensure the even distribution of body fluids. Then, an investigator placed adhesive electrodes at four distinct sites (Vallecillo-Bustos et al., 2024; Tinsley et al., 2022). The first proximal electrode was positioned on the participant’s posterior wrist, midway between the radial and ulnar processes. The second proximal electrode was placed on the participant’s anterior ankle, midway between the medial and lateral malleoli of the tibia and fibula. The two remaining distal electrodes were positioned approximately 5 cm below their corresponding proximal electrodes (Vallecillo-Bustos et al., 2024; Tinsley et al., 2022). After all electrodes were secured, a single measurement was performed and immediately assessed for quality by a trained investigator via visual inspection of Cole plots.

Quality assurance assessments were conducted each morning prior to testing using the manufacturer provided test-cell, and all testing was performed at a standard frequency of 50 kHz using the default sex and body density, proportion, and hydration coefficients suggested by the manufacturer (Moon et al., 2008). To simulate a level of standardization most likely to be employed outside of a research setting, participants were not required to void their bladder, and hydration status was not confirmed using urine specific gravity or an eight-point color chart (Graybeal et al., 2020) prior to testing. Instead, participants were simply instructed to remain hydrated leading up to the start of their overnight fast, as both bladder fluid volume and hydration status have shown to have minimal effect on BIA assessments when compared to standardization techniques (Ferri-Morales et al., 2024; Randhawa et al., 2021; Ritz and Source Study, 2001) that control for each component.

### 2.4 Smartphone 3D body scanning application

The procedures for obtaining automated anthropometric estimates using a 3D smartphone body scanning application, along with its precision and agreement with criterion body composition techniques, have been described in detail elsewhere (Graybeal et al., 2024b; Graybeal et al., 2022a; Graybeal et al., 2022b; Graybeal et al., 2022c; Graybeal et al., 2023c; Graybeal et al., 2024d; Tinsley et al., 2024). In summary, a 3D smartphone body scanning application (MeThreeSixty®, Size Stream LLC, Cary, NC, USA) was used to automate digital anthropometric estimates. Although this method can automate hundreds of anthropometric estimates through advanced digital imaging and machine learning techniques, we chose to use only the raw estimates for appendicular limb lengths and total and segmental body volumes for our analysis based on their well-established relationships with body fluid measurements (Nickerson et al., 2022; Nishimura et al., 2020). Furthermore, body shape and adiposity indices were calculated from our automated anthropometrics (described in Section 2.5) and used for analysis.

For testing, participants removed all external metal and accessories, wore only tight-fitting athletic clothing, and tied their hair up so that it was above their shoulder line. After entering the participant’s age, sex, height, and weight into the application, the smartphone was placed in a stationary tripod in a position confirmed by application’s internal quality assurance protocols. Participants were then instructed to stand on a foot guide positioned in front of a neutral-colored (grey) background at a standardized distance from the smartphone. Once positioned, a single image was captured using the smartphone’s front-facing camera while the participant performed two distinct poses, resulting in two 2D images that were subsequently used to generate the 3D avatar. For the first pose, participants faced the smartphone while automated verbal prompts instructed them to widen their feet and laterally raise their arms. For the second pose, participants turned to the side so that their shoulder faced the smartphone while automated verbal prompts instructed them to look forward, bring their feet together, and fully extend their arms with their hands flat against their lateral thighs. All images were collected in a room without external light (i.e., windows).

### 2.5 Model development

The methods used to develop the prediction models have been described in detail in prior publications and are summarized hereafter (Graybeal et al., 2024b). Least absolute shrinkage and selection operator (LASSO) regression procedures were employed to develop prediction equations for TBW and ECF using demographic and smartphone-generated anthropometric predictor variables (18 total predictors). Demographic predictor variables included age (yrs), height (cm), weight (kg), sex (male/female), race (White/Black/Asian), and ethnicity (Hispanic/non-Hispanic). Anthropometric predictor variables included lengths (cm) of the arms and outer legs, and volumes (cm^3^) of the whole-body, arms, legs, torso, and busts. To produce single estimates for analysis, right and left limb lengths were averaged, and right and left volumes of the arms, legs, and busts were summed. Additionally, a body shape index (ABSI) (Krakauer and Krakauer, 2012) and an appendage-to-trunk circumference index (ATI) (Harty et al., 2020) were included and calculated using the following equations:

$$ABSI=\frac{\text{waist}\;\text{circumference}}{{BMI}^{2/3}\times {height}^{1/2}}$$

$$ATI=\frac{L\;\text{upper}\;\text{arm}+R\;\text{upper}\;\text{arm}+L\;\text{thigh}+R\;\text{thigh}+L\;\text{calf}+R\;\text{calf}}{\text{stomach}\;\text{circumference}}$$

where all estimates represent smartphone-derived circumference (cm) estimates other than BMI (kg/m^2^) and height. Trunk-to-leg volume (TLV), which has also shown to be indicative of chronic disease (Bennett et al., 2024), was also included and calculated as the smartphone-derived trunk volume divided by the summed leg volume.

To formulate and cross-validate the new TBW and ECF prediction equations, a training dataset comprising 80% of the sample (n = 272) and a testing dataset consisting of the remaining 20% (n = 66) were generated using random sampling techniques (The R Project, 2024). The descriptive characteristics of the training and testing samples, as well as the combined sample (n = 338) are presented in Table 1. Using the 10-fold cross-validation and one SE rule (McCarthy et al., 2023) to identify the optimal λ value, LASSO regression was applied to fit models in the training dataset. This method works by identifying the predictor variables that reduce prediction error while simultaneously shrinking the coefficients of unnecessary variables towards zero, effectively omitting the variables from the model (McCarthy et al., 2023; Friedman et al., 2010; Tibshirani, 1996). This approach aims to produce the most parsimonious model, in addition to minimizing both multicollinearity and model overfitting. After the TBW and ECF prediction models were formulated using the training sample, the models were applied to the testing sample to predict each variable. Notably, smartphone-predicted ICF was calculated as the difference between the smartphone-predicted TBW and ECF estimates in the testing sample. Similar to recent investigations (Haynes et al., 2024), ICF was calculated (as opposed to predicted) to ensure that the summation of smartphone-derived ECF and ICF were equivalent with smartphone-predicted TBW.

**TABLE 1:** Descriptive characteristics of the combined, training, and testing samples.

	Combined sample	Training sample	Testing sample
N	338	272	66
Sex (F/M)	190/148	152/120	38/28
Race (W/B/A)	158/109/71	124/88/60	34/21/11
Ethnicity (H/NH)	16/322	11/261	5/61
Age (y)	23.8 ± 8.2	23.7 ± 8.1	24.3 ± 8.5
Height (cm)	169.0 ± 74.6	168.7 ± 9.7	170.0 ± 9.4
Weight (kg)	74.6 ± 19.9	74.8 ± 20.5	73.9 ± 17.4
BMI (kg/m^2^)	26.0 ± 5.8	26.1 ± 5.9	25.4 ± 5.1
Body fat (%)^a^	29.4 ± 8.5	29.5 ± 8.5	28.9 ± 8.7
Fat mass (kg)^a^	22.4 ± 11.0	22.6 ± 11.4	21.6 ± 9.3
Fat-free mass (kg)^a^	52.2 ± 12.9	52.2 ± 12.9	52.2 ± 12.9
TBW (L)^a^	38.3 ± 9.40	38.3 ± 9.4	38.2 ± 9.4
Predicted TBW (L)^b^	-	-	38.2 ± 7.4
ECF (L)^a^	16.0 ± 3.9	16.0 ± 4.0	15.9 ± 3.9
Predicted ECF (L)^b^	-	-	16.0 ± 3.2
ICF (L)^a^	22.3 ± 5.60	22.3 ± 5.6	22.3 ± 5.7
Predicted ICF (L)^b^	-	-	22.3 ± 4.2
ECF/TBW^a^	0.42 ± 0.02	0.42 ± 0.02	0.42 ± 0.02
Predicted ECF/TBW^b^	-	-	0.42 ± 0.01
ECF/ICF^a^	0.72 ± 0.06	0.72 ± 0.07	0.72 ± 0.06
Predicted ECF/ICF^b^	-	-	0.72 ± 0.03
Overhydration _ECF/TBW_ ^a^	115 (34.0%)	93 (34.2%)	22 (33.3%)
Predicted Overhydration _ECF/TBW_ ^b^	-	-	31 (47.0%)
Excess Fluid _ECF/ICF_ ^a^	83 (24.6%)	66 (24.3%)	17 (25.8%)
Predicted Excess Fluid _ECF/ICF_ ^b^	-	-	27 (40.9%)
Arm length (cm)	58.3 ± 3.6	58.2 ± 3.7	58.7 ± 3.2
Outer leg length (cm)	102.9 ± 5.4	102.7 ± 5.5	103.5 ± 5.3
Body volume (cm^3^)	72,689 ± 21,142	72,578 ± 21,763	72,407 ± 18,512
Arm volume (cm^3^)	7,848 ± 2,513	7,836 ± 2,542	7,900 ± 2,410
Leg volume (cm^3^)	18,880 ± 4,748	18,841 ± 4,905	19,040 ± 4,063
Torso volume (cm^3^)	45,882 ± 14,563	46,000 ± 15,017	45,395 ± 12,613
Bust volume (cm^3^)	773 ± 476	768 ± 488	796 ± 426
TLV (cm^3^)	2.41 ± 0.31	2.43 ± 0.31	2.37 ± 0.28
ABSI	0.79 ± 0.06	0.79 ± 0.06	0.79 ± 0.05
ATI	2.88 ± 0.15	2.87 ± 0.15	2.91 ± 0.14

Data are presented as mean ± standard deviation, N, or N (percent of the column total).

Appendicular and bust volumes were calculated as the sum of the right and left sides. For ATI, all variables were collected using smartphone-derived measurements.

^a^ estimates produced using bioelectrical impedance spectroscopy.

^b^ estimates produced using the new smartphone prediction model in the testing sample.

F: female; M: male; W: white; B: black; H: hispanic; NH: non-Hispanic; BMI: body mass index; TBW: total body water; ECF: extracellular fluid; ICF: intracellular fluid; TLV: trunk-to-leg volume; ABSI: a body shape index; ATI: appendage-to-trunk circumference index.

### 2.6 Statistical analyses

The smartphone-predicted body fluid estimates were evaluated against those produced by the criterion BIS in the testing sample using various agreement analyses, including null hypothesis significance tests, equivalence tests, coefficients of determination (*R* ^2^), root mean squared error (RMSE), standard error of the estimate (SEE), concordance correlation coefficients (CCC), and Bland-Altman and Deming regression analyses. For equivalence testing, equivalence regions were defined as ±1.0 L for TBW using two-sided TOST tests (Dixon et al., 2018). Given that ECF and ICF constitute approximately 40% and 60% of TBW, respectively, ECF equivalence regions were defined as ±0.40 L, and ICF equivalence regions were defined as ±0.60 L (Bhave and Neilson, 2011). The 95% limits of agreement (LOA) were determined using Bland-Altman analyses, and linear regression techniques were used to evaluate proportional bias. Agreement between the smartphone-predicted body fluid estimates and the line-of-identity from Deming regression was confirmed if the 95% confidence intervals for the intercept and slope contained the values 0 and 1, respectively. Mean difference (MD) between methods was calculated as the smartphone-predicted body fluid estimates minus the estimates produced by BIS (reference method).

Considering the significant clinical implications of assessing fluid overload and imbalance in the context of cardiovascular, renal, and hepatic abnormalities (Hara et al., 2009; Nongnuch et al., 2015; Accardi et al., 2021; Horino et al., 2023; Kim et al., 2017; Park et al., 2018), we also evaluated the ability of the retained smartphone variables to accurately predict fluid overload and imbalance classifications in the testing sample. Using the estimates produced by BIS, fluid overload was classified as having an ECF/TBW ≥0.43, and fluid imbalance was classified as having an ECF/ICF ≥0.76. Notably, these cutoffs correspond to the 75th percentile values for these measurements in our training sample, and align with previously reported values (Chamney et al., 2007). A receiver operating characteristic (ROC) curve analysis was used to identify the optimal cutoff values for both ECF/TBW (cutoff: ≥0.30) and ECF/ICF (cutoff: ≥0.25), defined as the point at which the sum of the sensitivity and specificity was maximized (Park et al., 2018). Then, the ability of the retained smartphone variables to accurately predict fluid overload and imbalance classifications was evaluated using binomial logistic regression. Although the final TBW and ECF prediction models retained different variables (see Section 3), all predictor variables from both models were included in each logistic regression analysis, as both ECF/TBW and ECF/ICF are predicted using the outputs of each smartphone prediction model. Specifically, calculating the smartphone-derived ECF/TBW ratio requires the use of both models. Similarly, determining the ECF/ICF also necessitates both models, as ICF is derived from the difference between smartphone-predicted TBW and ECF. The positive and negative proportions of the smartphone-predicted fluid overload and imbalance were compared to the proportions determined by BIS using the ROC area under the curve (AUC), chi-square tests with corrections for continuity, R^2^_McFadden_, and sensitivity, specificity, accuracy, and positive (LR+) and negative (LR-) likelihood ratios. Acceptable accuracy of the smartphone-predicted classifications were defined as having an AUC ≥0.70 (Mandrekar, 2010) or having a summed sensitivity and specificity of ≥1.50 (Power et al., 2013).

## 3 Results

The coefficients of the variables retained in both the TBW and ECF models are presented in Supplementary Table S1. For TBW, the retained variables included sex, race (Asian), height, weight, arm volume, ABSI, and TLV, which produced following prediction model:

$$\begin{array}{cc} Total\;body\;water\;\left(L\right) & =1.9547+2.3819\left(female=0;male=1\right)\\ & \;-1.0516\left(0=non‐Asian;1=Asian\right)\\ & \;+0.1714\left(height\right)+0.2243\left(weight\right)\\ & \;+0.0004\left(arm\;volume\right)-18.9566\left(ABSI\right)\\ & \;+0.5808\left(trunk‐to‐leg\;volume\right) \end{array}$$

For ECF, retention of the variables age, sex, height, weight, arm volume, and ABSI resulted in the following model:

$$\begin{array}{cc} Extracellular\;fluid\;\left(L\right)= & -5.9009+0.0007\left(age\right)+1.9928\\ & \;\left(female=0;male=1\right)\\ & \;+0.0832\left(height\right)+0.1057\left(weight\right)\\ & \;+0.00005\left(arm\;volume\right)-1.7374\left(ABSI\right) \end{array}$$

All variance inflation factors and tolerance statistics were ≤5.77 and ≥0.173, respectively, when evaluated in the training sample.

**FIGURE 1:**
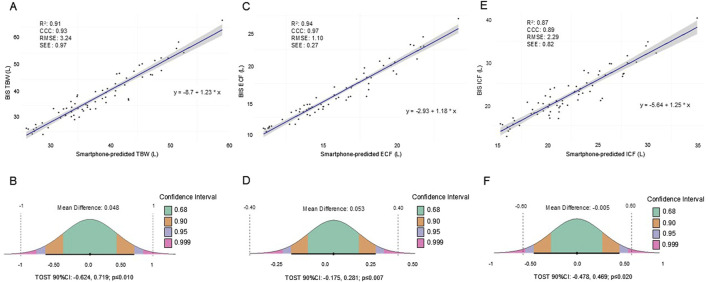
**(A-F)**. Simple regression **(A,C,E)**, and equivalence plots **(B,D,F)** demonstrating the agreement between smartphone-predicted and BIS TBW, ECF, and ICF in the testing sample (n = 66). For the simple regression plots, the solid blue line and its corresponding shaded area represents the regression line and its 95%CI, respectively. For the equivalence plots, the average MDs and TOST 90%CIs are presented, where the colored regions represent the TOST CIs displayed in the CI legend, the black circles and intersecting horizontal lines represent the MD and the TOST 90%CIs, respectively, and the vertical dashed lines indicate the equivalence regions. BIS: bioelectrical impedance spectroscopy; CCC: concordance correlation coefficient; CI: confidence interval; ECF: extracellular fluid; ICF: intracellular fluid; MD: mean difference calculated as the smartphone-predicted fluid estimate minus the actual estimate produced by BIS; *R* ^2^: coefficient of determination; RMSE: root mean square error; SEE: standard error of the estimate; TBW: total body water; TOST: values from the TOSTER package in R.

**FIGURE 2:**
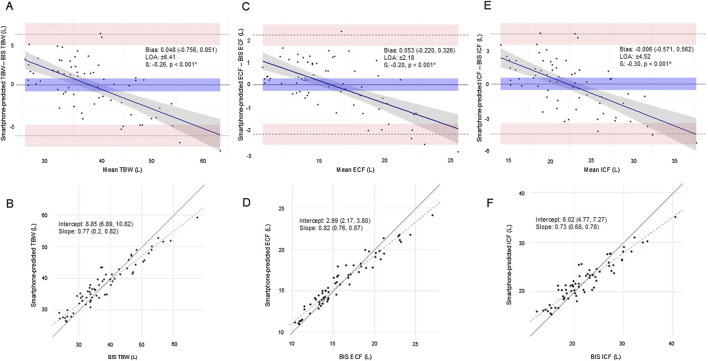
**(A-F)**. Bland-Altman **(A,C,E)** and Deming regression **(B,D,F)** plots demonstrating the agreement between smartphone-predicted and BIS TBW, ECF, and ICF in the testing sample (n = 66). For the Bland-Altman plots, the upper and lower dashed lines represent the 95% LOA, the middle-dashed line represents the MD (bias) between the smartphone-predicted fluid estimate and the measurement produced by BIS, and the solid blue line represents the regression line. The shaded areas within each Bland-Altman plot represent the 95%CI for each intersecting dashed line. For the Demming regression plots, the solid black line represents the line of identity and the red dashed line represents the regression line. β: proportional bias coefficient; BIS: bioelectrical impedance spectroscopy; CI: confidence interval; ECF: extracellular fluid; ICF: intracellular fluid; LOA: 95% limits of agreement; MD: mean difference calculated as the smartphone-predicted fluid estimate minus the actual estimate produced by BIS; TBW: total body water. * statistically significant at p < 0.050.

Figures 1A–F shows linear regression and equivalence tests demonstrating the agreement between the TBW, ECF, and ICF estimates produced by the smartphone prediction model and those measured using BIS, while Figures 2A–F presents the agreement between each method using Bland-Altman and Deming regression analyses. All body fluid estimates produced using the smartphone application (TBW_MD_: 0.048; ECF_MD_: 0.053; ICF_MD_: 0.006) demonstrated equivalence with BIS (Figures 1B,D,F; all TOST 90%CI: p ≤ 0.020) and null hypothesis significance tests revealed no significant differences between methods (Figures 2A,C,E; all p ≥ 0.701). *R* ^2^ (0.87-0.94), CCC (0.89-0.97), RMSE (1.10-3.24 L), and SEE (0.27-0.97 L) values revealed excellent agreement between methods (Figures 1A,C,E), though proportional biases (Figures 2A,C,E; all p < 0.001), moderate-to-large 95% LOA (Figures 2A,C,E; TBW: ±6.41 L; ECF: ±2.18 L; ICF: ±4.52 L) and differences from the line-of-identity were observed (Figures 2B,D,F).

**FIGURE 3:**
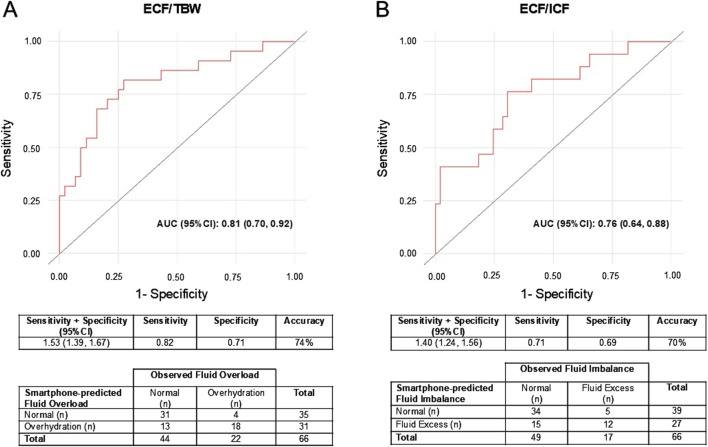
**(A, B)**. ROC curves and corresponding AUCs demonstrating the ability of the predictor variables retained during LASSO regression to predict fluid overload **(A)** and imbalance **(B)** in the testing sample (n = 66). Fluid overload was classified as having an ECF/TBW ≥0.43, and fluid imbalance was classified as having an ECF/ICF ≥0.76, which correspond to the 75th percentile values for these measurements in our training sample. Using the measurements produced by BIS, an ROC curve identified the optimal cutoff values, defined as the point at which the sum of the sensitivity and specificity was maximized, for both ECF/TBW (cutoff: ≥0.30) and ECF/ICF (cutoff: ≥0.25). Positive and negative cases are presented for both smartphone-predicted and BIS fluid overload and imbalance, as well as the AUC (acceptable performance = ≥0.70) sensitivity, specificity, joint sensitivity and specificity (acceptable performance = ≥1.50), and accuracy of the smartphone-predicted fluid estimates. 95%CI: 95% confidence intervals; AUC: area under the curve; BIS: bioelectrical impedance spectroscopy; ECF: extracellular fluid; ICF: intracellular fluid; LASSO: least absolute shrinkage and selection operator; ROC: receiver-operating characteristic; TBW: total body water.

Figures 3A,B illustrates the accuracy of detecting fluid overload and imbalance using the retained smartphone predictor variables. The smartphone prediction models revealed a prevalence of 47.0% (n = 31) for overhydration and 40.9% (n = 27) for fluid excess, overestimating the 33.3% (n = 22) and 25.8% (n = 17) prevalence determined by BIS, respectively. While chi-square tests were significant for both fluid overload (χ^2^: 14.1, p < 0.001) and imbalance (χ^2^: 6.8, p = 0.009), both model AUCs (overload [95%CI]: 0.81 [0.70, 0.92]; imbalance [95%CI]: 0.76 [0.64, 0.88]) exceeded the 0.70 threshold, indicating acceptable model performance. Although the diagnostic accuracies of smartphone-predicted fluid overload (Figure 3A; 74%) and imbalance (Figure 3B; 70%) were similar, only smartphone-predicted fluid overload met the threshold for summed sensitivity and specificity of 1.50 (Figures 3A,B; overload [95%CI]: 1.53 [1.39, 1.67]; imbalance [95%CI]: 1.40 [1.24, 1.56]).

## 4 Discussion

BIA is becoming increasingly popular in clinical settings. This is particularly true for raw bioelectrical impedance parameters, which are used to monitor shifts in cellular health in many chronic disease states (Mattiello et al., 2020). However, recent findings indicate that raw impedance information may having conflicting associations with chronic disease risk factors (Graybeal et al., 2024c; Oliveira et al., 2022; Bučan Nenadić et al., 2022), resulting in a lack of clinical confidence in these measurements. Given the inconsistent findings for the expanded use of BIA, traditional body fluid and distribution estimates remain crucial for tracking the dynamic fluid shifts that mirror many chronic diseases. However, there are inherent barriers to the widespread use of BIA (discussed in Section 1.). To address the concerns with existing methods, our study aimed to determine whether the automated anthropometrics produced by a smartphone scanning application could be adapted to provide accurate predictions of body water and fluid distribution, as well as distinguish more clinically relevant measures such as fluid overload and imbalance. This is the first study, to our knowledge, to demonstrate that all body fluid estimates predicted by a newly-developed smartphone prediction model showed excellent agreement with those produced by BIS and revealed acceptable accuracy in classifying fluid overload and imbalance. This advancement further expands the utility of this technique, addressing the critical need for mobile and remote health assessment options in modern healthcare systems.

Body volumes obtained from smartphone scanning applications have previously shown to be a reliable body volume replacement method for underwater weighing and DXA, and have demonstrated acceptable accuracy when applied to multicompartment models (Fedewa et al., 2021; Sullivan et al., 2022). Given that BIS produces volumetric fluid estimates, and considering the overlap between body volume (shown to be accurately assessed using smartphone anthropometry) and body water, it is unsurprising that this smartphone scanning application exhibited excellent accuracy in predicting body fluid. Since ECF and ICF are encapsulated in total body water (TBW) estimates, often within a narrow range, it is also unsurprising that the fluid compartment estimates derived from smartphone-based body volumes showed excellent agreement. With the addition of our newly-developed TBW prediction model, smartphone scanning applications can now accurately quantify multicompartment body composition estimates (Graybeal et al., 2022a; Harty et al., 2020), as well as each major body composition compartment independently (TBW, BMC, FM, body volume). Given the continual advancement of mobile anthropometry, the increasing preference for mobile health tools, and the joint and standalone relevance of these measurements in both research and clinical practice, mobile applications may soon be integrated into routine and remote health screening procedures.

Though smartphone-derived body volumes were our primary variables of interest, other variables were retained that are worthy of further discussion. Notably, our final models included indices of ABSI, which estimates WC relative to body stature, and TLV, which represents the volume of the torso relative to the summed volume of the legs. Interestingly, ABSI (Dhana et al., 2016) and TLV (Wilson et al., 2013) have shown to be positively and negatively associated with fat mass (FM) and FFM, respectively—associations often used to indicate sarcopenic obesity. Given that the largest proportion of FFM consists of ICF, which overlaps with skeletal muscle mass (Serra-Prat et al., 2019), it is unsurprising that greater ABSI resulted in lower TBW concentrations in our study. Supporting these findings, ABSI has previously been retained in smartphone BMC prediction models, where higher ABSI resulted in lower BMC estimates (Graybeal et al., 2024b). The skeletal muscle and bone reflect a singular system (i.e., musculoskeletal) (Battafarano et al., 2020), where forces exerted by the muscle are required to effectively remodel the bone (Graybeal et al., 2024b). Therefore, if higher ABSI indicates lower FFM, its consistent inclusion in models predicting variables highly related to skeletal muscle is expected. Our findings for TLV contradicted these results, as greater TLV resulted in greater TBW. When combined with arm volume, TLV accounts for the volumes of each major body segment, potentially providing a more mathematically parsimonious model for predicting whole-body estimates such as TBW. However, unlike ABSI, TLV was not retained in the ECF model. This may be because TLV does not adequately represent ECF, often used to indicate fluid retention and disease risk, in a younger, healthier sample like our own, which is at a lower risk of developing discernible lower-leg edema. Importantly, ABSI and TLV serve as indirect indicators of body composition and require careful consideration when used for clinical inference.

Predicting raw body fluid estimates from a smartphone scanning application has broad applicability and improves clinical flexibility, but these continuous estimates may provide little value as a prognosticator of disease risk when clinical decision-making most often relies on discrete diagnostic cutoffs. Therefore, our finding that smartphone anthropometrics could accurately identify fluid overload and imbalance classifications may have several clinical implications. For instance, BIA is not typically implemented during routine care outside of specialized healthcare settings or fitness facilities and requires frequent assessments (sometimes within a single day) that present additional constraints for both patients and staff. Due to the limited alternative assessment methods, early-stage fluid imbalances often go undiagnosed, leading to disease progression that eventually necessitates more rigorous testing. Given the simplicity of our newly-developed smartphone scanning model, this technique could be easily implemented as an early screening tool at point-of-care, with the potential to better inform preventative measures before disease progression or the need for more comprehensive cardiorenal and hepatic health assessments. Moreover, the remote capability of this technique increases the ease of continual monitoring, eliminating the need for frequent or prolonged office visits that are often burdensome for both patients and practitioners. However, while the performance of our models was adequate, the minor discrepancies between methods warrants consideration when employed as a screening tool or when used in clinical decision-making. At present, further follow-up testing is still recommended.

There are a few notable limitations of this study that warrant discussion. Swim caps, which are often recommended during whole-body volume assessments, were not worn during the smartphone scanning assessments. However, this likely represents the assessment attire that would be used in practice and does not impact the segmental volume estimates that were retained in our final prediction models. Our study sample was primarily young and apparently healthy, limiting our ability to generalize findings to more clinical populations at the greatest risk of cardiovascular, renal, or hepatic diseases. However, the aim of our study was to provide proof-of-concept for the ability of this smartphone scanning application to evaluate body fluids and imbalances. Including individuals with known diseases experiencing more dynamic day-to-day fluid shifts or undergoing treatments (including the use of prescription medication to manipulate body fluids) may have inhibited our ability to provide such evidence. It should be noted that, at present, smartphone-based assessments cannot be extrapolated to older or more clinical populations without further validation and should be used as a screening tool that informs further testing. Those at the greatest risk of cardiovascular, kidney, or renal disease should undergo more thorough testing provided by a healthcare professional until further evaluation of this technique confirms its diagnostic ability (Graybeal et al., 2024b). We did not perform repeated scans for BIS nor the smartphone scanning application, so we are unable to demonstrate the repeatability of either technique. However, BIS (Brandner et al., 2022; Siedler et al., 2023; Looney et al., 2024) and the smartphone application (Graybeal et al., 2022b; Graybeal et al., 2023c; Smith et al., 2022) have well-established precision and reliability. Proportional biases and LOA were large, potentially limiting clinical applicability; however, large limits of agreement and proportional biases are common due to systemic differences between methodologies. So, while these biases do not completely invalidate this technique, users should interpret results with caution. Although stringent model development techniques that limit multicollinearity were performed, it is possible that this model performs differently in alternate samples due to its specificity to the developmental population. Therefore, while we cannot guarantee its performance in external groups that significantly differ from our training sample, we showed excellent performance of the model during cross-validation (and low multicollinearity), which should improve confidence in its application. Finally, anthropometrics collected by various smartphone applications may differ from those used in our study and should not be used interchangeably within our newly-developed prediction model.

In conclusion, this study demonstrated that that all body fluid estimates produced by our smartphone prediction model showed excellent agreement with those predicted by BIS and revealed acceptable accuracy when classifying cutoffs for fluid overload and imbalance. Given the expansion of this technique, smartphone body scanning applications demonstrate promise as a clinically meaningful body fluid assessment tool that addresses the growing need for mobile and remote health assessments. As digital anthropometry continues to advance, there may soon be a time when this automated method replaces the need for highly-trained technicians/specialists and larger, more sophisticated equipment for anthropometric evaluation in routine healthcare. Although our models demonstrated excellent agreement, further research in external samples is required before broad implementation. Because the findings of our study are preliminary, they should not be used to guide clinical decisions, and those choosing to employ these models should do so with caution until future research can confirm our findings.

## Data Availability

The raw data supporting the conclusions of this article will be made available by the authors, without undue reservation.
