# Supplementary material for: A predictive model for body water and fluid balance using 3D smartphone anthropometry
Source: Front Physiol. 2025 Jun 23;16:1577049. doi: 10.3389/fphys.2025.1577049 (PMC12230031; doi:10.3389/fphys.2025.1577049)
Supplement: Supplementary file 1 [file DataSheet1.PDF]

Supplemental Table 1. Model coefficients predicting total body water and extracellular fluid from the smartphone anthropometrics retained during LASSO regression

Retained predictor variables	Total body water (L)	Extracellular fluid (L)
Intercept	1.9547	-5.9009
Age (y)	0	0.0007
Sex	2.3819	1.9928
Asian	-1.0516	0
Height (cm)	0.1714	0.0832
Weight (kg)	0.2243	0.1057
Arm volume (cm^3)	0.0004	0.00005
ABSI ($m^{11/6}/kg^{-2/3}$)	-18.9566	-1.7374
TLV (cm^3)	0.5808	0

All data are presented as the unstandardized coefficients for each variable within the respective body fluid model. Coefficients retained in either the TBW or ECF model, but not both, are included in each column, showing a coefficient of 0 for the model in which the variable was not retained. The coefficients of all other predictor variables were shrunk to "0" for both models and are therefore not included in the final model equations or the table.

"Sex" was defined as 0 = female and 1 = male. Asian was defined as 0 = not Asian and 1 = Asian. Arm volume was calculated as the sum of the left and right arm volumes. ABSI was calculated as: smartphone-derived waist circumference ÷ ($BMI^{2/3}$ x $height^{1/2}$); TLV was calculated as: smartphone-derived trunk volume ÷ smartphone left + right leg volume.

ABSI: a body shape index; TLV: trunk-to-leg volume.
